# THE EFFECT OF PERCEIVED APPRECIATION ON CAREGIVER WELL-BEING: A TEST OF EQUITY THEORY

**DOI:** 10.1093/geroni/igz038.2667

**Published:** 2019-11-08

**Authors:** Kylie Meyer, Frank Puga, Carolyn E Pickering

**Affiliations:** University of Texas Health Science Center San Antonio, San Antonio, Texas, United States

## Abstract

Equity theory suggests that equal (reciprocal) exchange of support in social relationships leads to better outcomes for members. However, in caregiving, exchange of support may become unbalanced due to heightened instrumental support from caregivers (CG). Imbalance may be exacerbated in dyads where the care recipient (CR) has dementia, since cognitive changes can attenuate expression of social support. One way social support is demonstrated in through show of appreciation. We used data from National Study on Caregiving (NSOC) to test whether CGs who did not feel appreciated by CRs were more likely to experience depression in the future. To accomplish this, we applied lagged dependent variable (LDV) models to the 2011 and 2015 NSOC waves (N=150). CGs who felt appreciated by the CR in the 2011 wave had 0.22 times the odds of being depressed in 2015 as those who did not feel appreciated by the CR (CI 0.07 to 0.68). This effect appears to exist primarily among CGs to people with dementia. In stratified models, we found CGs to people with dementia in 2015 had 0.04 times the odds of being depressed in 2015 if they previously felt appreciated by the CR (CI 0.003 to 0.40). A statistically significant effect was not observed among those assisting someone without depression (OR=0.65, CI 0.22 to 1.91). Results suggest that CGs to people with dementia who feel appreciated have less risk of experiencing depression. Findings have applications for future interventions for caregiver wellbeing, such as enhancing perceived appreciation (e.g., cognitive restructuring, communication training).

